# Data on processing of Ti-25Nb-25Zr β-titanium alloys via powder metallurgy route: Methodology, microstructure and mechanical properties

**DOI:** 10.1016/j.dib.2018.01.093

**Published:** 2018-02-03

**Authors:** D. Ueda, G. Dirras, A. Hocini, D. Tingaud, K. Ameyama, P. Langlois, D. Vrel, Z. Trzaska

**Affiliations:** a Department of Mechanical Engineering, Ritsumeikan University, Kustasu, Shiga, Japan; b Université Paris 13, LSPM-CNRS, 99 avenue Jean-Baptiste Clément, 93430 Villetaneuse, France

**Keywords:** Titanium alloys, Spark plasma sintering, Harmonic structure, Cyclic shear

## Abstract

The data presented in this article are related to the research article entitled “Cyclic Shear behavior of conventional and harmonic structure-designed Ti-25Nb-25Zr β-titanium alloy: Back-stress hardening and twinning inhibition” (Dirras et al., 2017) [1]. The datasheet describes the methods used to fabricate two β-titanium alloys having conventional microstructure and so-called harmonic structure (HS) design via a powder metallurgy route, namely the spark plasma sintering (SPS) route. The data show the as-processed unconsolidated powder microstructures as well as the post-SPS ones. The data illustrate the mechanical response under cyclic shear loading of consolidated alloy specimens. The data show how electron back scattering diffraction(EBSD) method is used to clearly identify induced deformation features in the case of the conventional alloy.

**Specifications Table**

**Table:** 

Subject area	*Physics*
More specific subject area	*Physical metallurgy*
Type of data	*Images, figures*
How data was acquired	*Powder metallurgy, SPS (Sumiseki Materials Co., Ltd: DR. SINTER SPS-1030), SEM (Carl Zeiss Supra 40 VP-FEG), EBSD (TexSEM OIM 5 Software, simple shear cyclic test (home-made device mounted on an MTS M20 testing machine)).*
Data format	*Analyzed*
Experimental factors	*Ti-25Nb-25Zr powders were prepared by plasma rotating electrode method. The Ti-25Nb-25Zr electrode was prepared from a commercial bar.*
Experimental features	*For HS design, the powder was ball-milled. Then obtained powders were sintered by SPS. Specimens for the evaluation of the mechanical properties were machined from the obtained compacts and tested at room temperature under cyclic shear loading conditions.*
Data source location	*LSPM-CNRS, université Paris 13, Villetaneuse, France; Ameyama Lab, Dpt of Mechanical Engineering, Ritsumeikan University, Kusatsu-Shiga, Japan*
Data accessibility	*With this article*

**Value of the data**

• The data are related to a new approach that uses powder metallurgy route combined with severe plastic deformation to process bulk and dense materials with a specific bimodal-like design.
• The concept described in the datasheet can be used to process a 3D network of ultrafine grains enclosing coarse grains. It can be applied to various metals and alloys.
• The data may be useful in comparing the mechanical behavior and properties under cyclic shear loading of heterogeneous (bimodal-like) microstructures obtained via conventional routes.
• The data show how EBSD investigations are used to identify the nature of mechanical twins in a β-Titanium alloy.

## 1 Data

β-titanium Ti-25Nb-25Zr alloys have been fabricated using SPS route. Two microstructures were obtained having conventional (homogeneous) and so-called harmonic structure (obtained after ball milling of the same powder). Initial powder microstructures, X-ray data of obtained compacts are presented. Stress-strain plots following simple shear cyclic tests are provided and scanning electron microscopy (SEM) images of as-processed microstructures and post-mortem EBSD investigations following simple shear cyclic tests are shown.

## 2 Experimental design, materials and methods

**Fig. 1:**
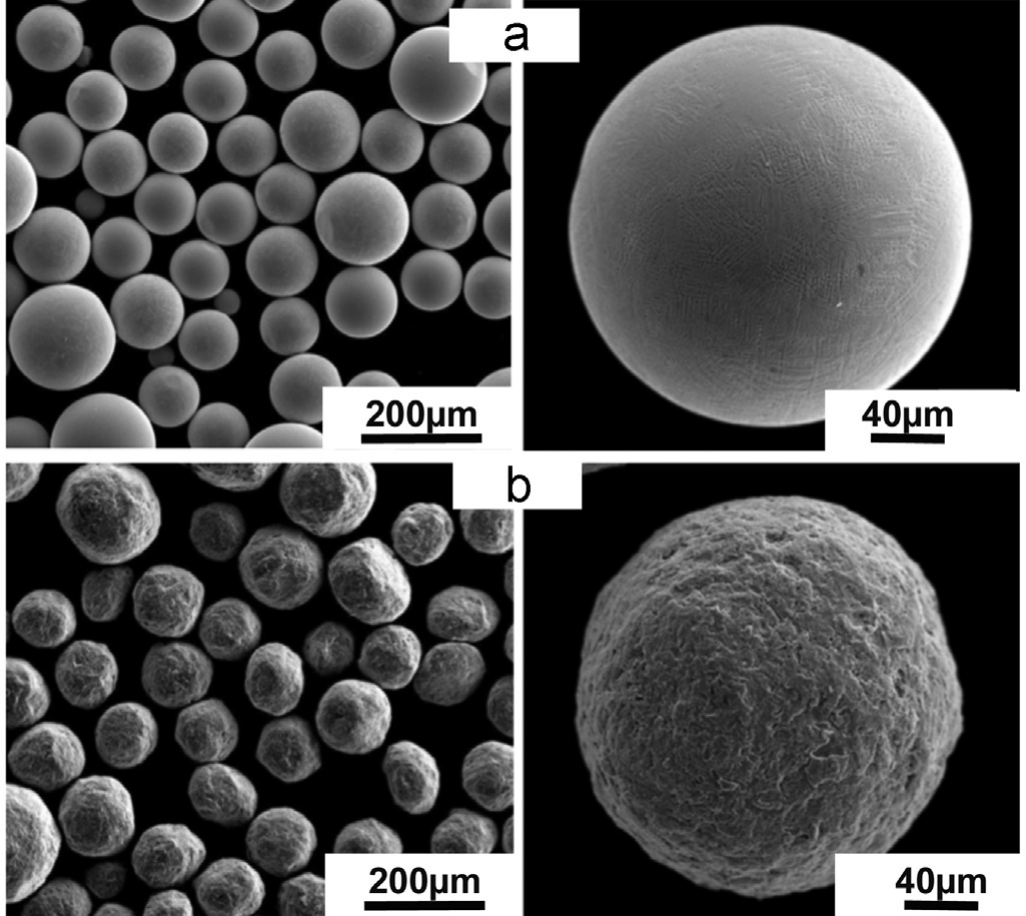
SEM images of the initial powders. (a) as-PREP and (b) ball-milled β-titanium Ti-25Nb-25Zr alloys, respectively.

**Fig. 2:**
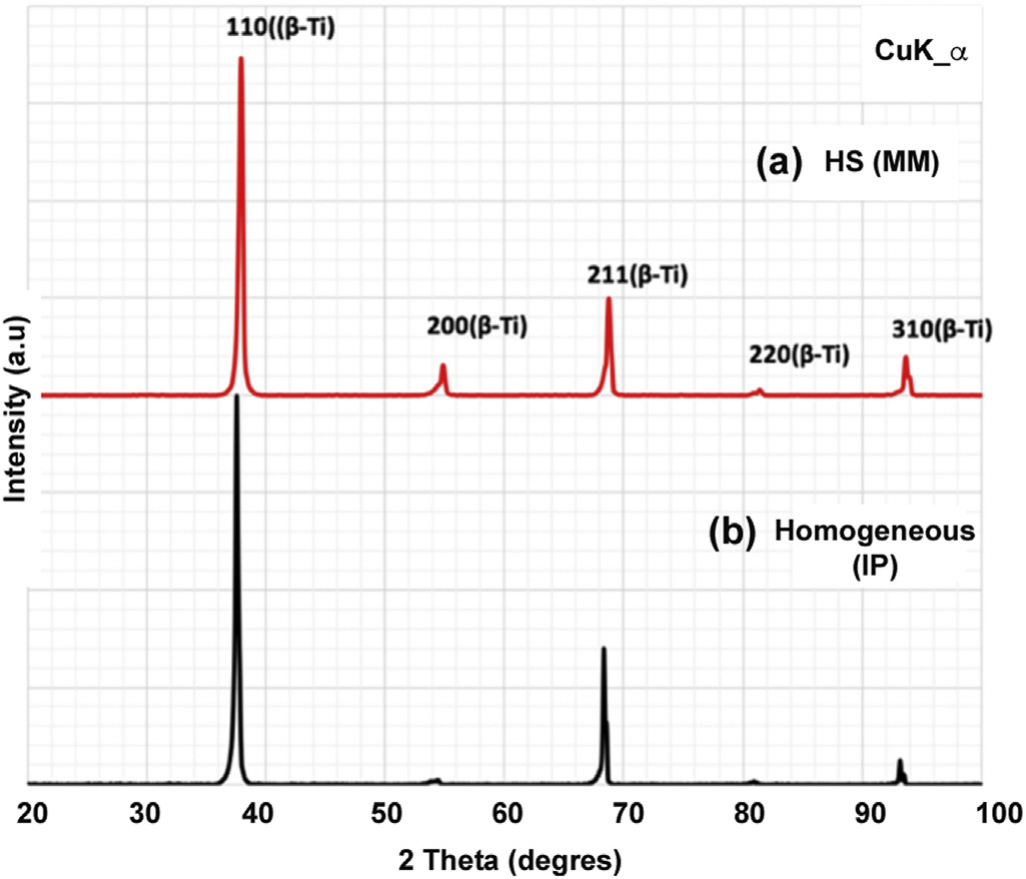
XRD patterns of the sintered compacts. (a) conventional and (b) HS β-titanium Ti-25Nb-25Zr alloys, respectively.

**Fig. 3:**
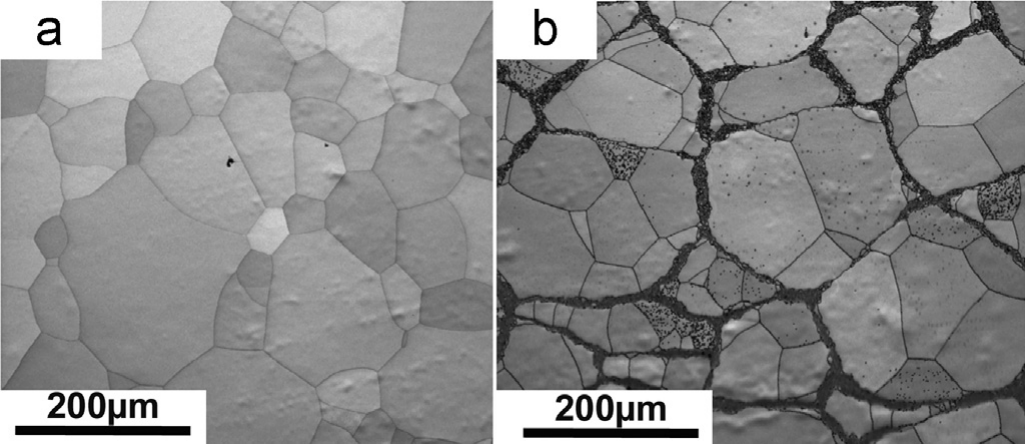
SEM images of the microstructures of (a) conventional and (b) HS Ti-25Nb-25Zr β-titanium alloys obtained after SPS.

As described in [1], Plasma Rotating Electrode Process (PREP) was used to prepare β-titanium Ti-25Nb-25Zr powders used to prepare both conventional (homogeneous) and harmonic-designed structure. Additional ball-milling step was used for the latter. Figs. 1a and b shows SEM images of the initial powders used for processing of homogeneous and HS microstructures, respectively. After milling, the surface of the powder was severely deformed. Controlling the amount of stored energy via adequate milling conditions allows for the design of the HS during sintering [2], [3], [5]–5]. Further, to obtain the corresponding homogeneous and HS alloy compacts, the powders were sintered using Dr Sinter (Japan) SPS apparatus. The sintering conditions are reported in [1]. Fig. 2 shows the X-ray diffraction patterns (XRD) of the compacted samples using a CuK-α radiation (*λ* = 0.1541 nm) for both alloys. Data shows only peaks corresponding to a β-crystalline structure. Fig. 3 shows SEM images of the as-sintered microstructures of conventional (Fig. 3a) and HS (Fig. 3b) compacts, respectively. The β-titanium Ti-25Nb-25Zr HS alloy displays a microstructure that consists in a 3D network of ultrafine-grained shell surrounding multi-crystalline cores.

**Fig. 4:**
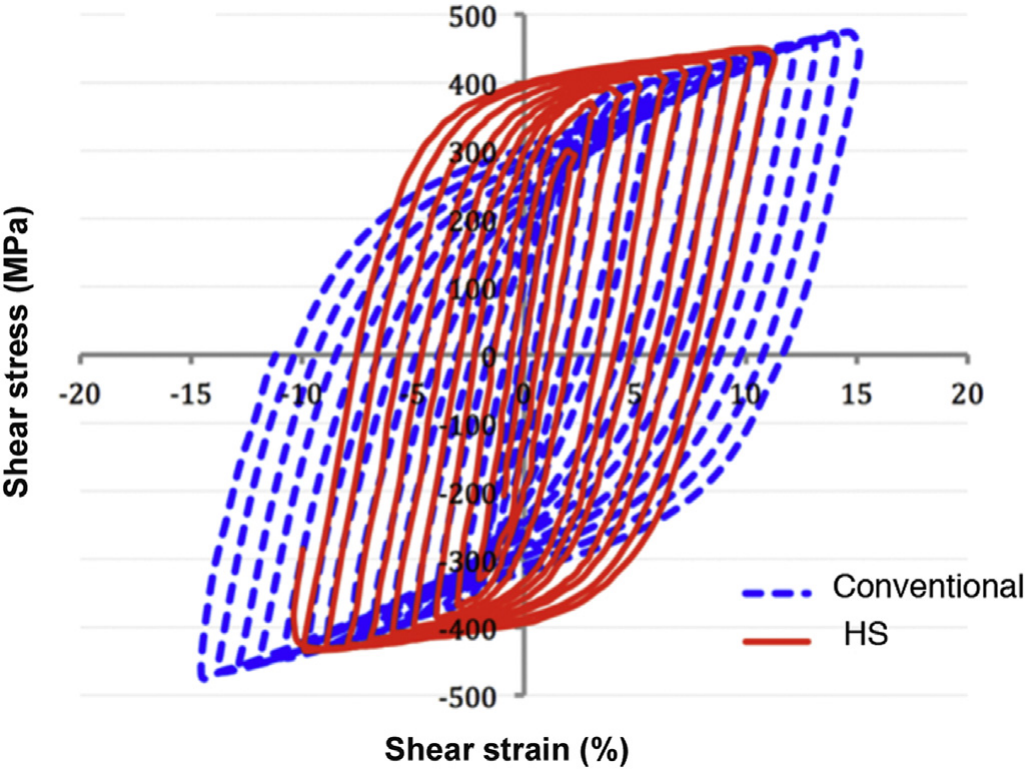
Cyclic test behavior of conventional (blue line) and HS (red line) Ti-25Nb-25Zr β-titanium alloys, respectively.

The mechanical properties were evaluated by simple shear cyclic tests performed on an MTS M20 testing machine equipped with a shearing device with a load capacity of 100 kN and using a constant strain rate of 10^−3^ s^−1^. The sample geometry was 20 mm in diameter and 1 mm in thickness with 15 × 2 × 1 mm^3^ sheared volume. The shear amplitude is incremental (by step of *ε* = ± 1%). Fig. 4 compares the behavior of both conventional (blue dashed line) and HS (red plain line) specimens. A detailed description is given in [1].

**Fig. 5:**
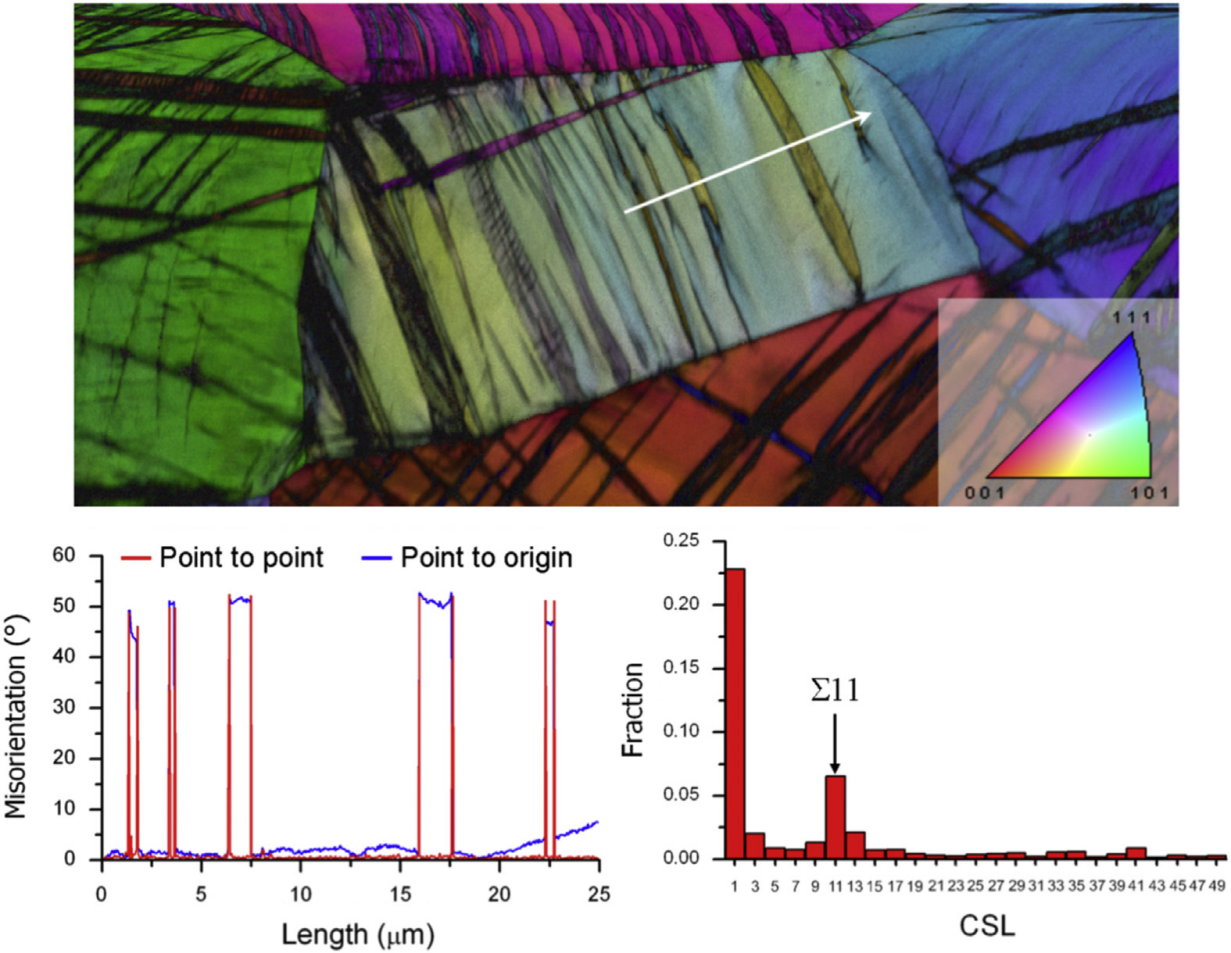
EBSD data analysis of the homogeneous sample after cyclic shear test. {332} <113> mechanical twins are identified.

Post-mortem microstructure investigations were carried out. Only the case of cyclically-deformed β-titanium homogeneous alloy is presented here. The corresponding EBSD data analysis is shown in Fig. 5. The data show a deformation substructure dominated by a high density of {332} <113> mechanical twins along with secondary twinning in between. A line scan (top image, white arrow) is used to compute the misorientation across the boundaries. An average misorientation of about 50° across boundaries is found as shown in the bottom left image. These boundaries are identified as Σ11 {332} <113> twin boundaries [6], [7], [8], [9].
